# Efficacy of Body Armor in Protection Against Blast Injuries Using a Swine Model in a Confined Space with a Blast Tube

**DOI:** 10.1007/s10439-021-02750-x

**Published:** 2021-03-08

**Authors:** Yasumasa Sekine, Daizoh Saitoh, Yuya Yoshimura, Masanori Fujita, Yoshiyuki Araki, Yasushi Kobayashi, Hitomi Kusumi, Satomi Yamagishi, Yuki Suto, Hiroshi Tamaki, Yosuke Ono, Toshiharu Mizukaki, Manabu Nemoto

**Affiliations:** 1 Division of Traumatology, Research Institute, National Defense Medical College (NDMC), 3-2 Namiki, Tokorozawa, 359-8513 Japan; 2 Dept. of Traumatology and Critical Care Medicine, NDMC, 3-2 Namiki, Tokorozawa, 359-8513 Japan; 3 Division of Environmental Medicine, Research Institute, NDMC, 3-2 Namiki, Tokorozawa, 359-8513 Japan; 4 Dept. of Defense Medicine, NDMC, 3-2 Namiki, Tokorozawa, 359-8513 Japan; 5 Dept. of Anatomy, NDMC, 3-2 Namiki, Tokorozawa, 359-8513 Japan; 6 Dept. of Military Nursing, NDMC, 3-2 Namiki, Tokorozawa, 359-8513 Japan; 7 Division of Graduate School, Dept. of Academic Affairs, NDMC, 3-2 Namiki, Tokorozawa, 359-8513 Japan; 8 Department of General Medicine, NDMC, 3-2 Namiki, Tokorozawa, 359-8513 Japan; 9 Military Medicine Research Unit, Test and Evaluation Command, Japan Ground Self Defense Force, 1-2-24 Ikejiri, setagaya-ku, Tokyo, 154-0004 Japan; 10 Dept. of Aeronautics and Astronautics, Tokai University, 4-1-1 Kitakaname, Hiratsuka, Kanagawa 259-1292 Japan; 11 Dept. of Emergency and Trauma Care, Saitama Medical University International Medical Center, 1397-1 Yamane, Hidaka, Saitama 350-1298 Japan

**Keywords:** Shock wave, Armor protection, Neurological reflex, Respiratory arrest, blast lung

## Abstract

The purpose of this study was to clarify whether or not body armor would protect the body of a swine model using a blast tube built at National Defense Medical College, which is the first such blast tube in Japan. Seventeen pigs were divided into two groups: the body armor group and the non-body armor group. Under intravenous anesthesia, the pigs were tightly fixed in the left lateral position on a table and exposed from the back neck to the upper lumbar back to the blast wave and wind with or without body armor, with the driving pressure of the blast tube set to 3.0 MPa. When the surviving and dead pigs were compared, blood gas analyses revealed significant differences in PaO2, PaCO2, and pH in the super-early phase. All pigs injured by the blast wave and wind had lung hemorrhage. All 6 animals in the body armor group and 6 of the 11 animals in the control group survived for 3 hours after injury. Respiratory arrest immediately after exposure to the blast wave was considered to influence the mortality in our pig model. Body armor may have a beneficial effect in protecting against respiratory arrest immediately after an explosion.

## Introduction

In recent years, terrorism-related bombings have become more frequent in the world, and the number of casualties from explosions has increased significantly.^2^,^19^,^29^,^31^,^32^ More than sixty thousand US soldiers have been killed or injured during wartime in Iraq and Afghanistan.^20^ Fortunately, there have been few terrorist bombings in Japan; however, Japan should not underestimate the risk of a bomb attack^1^ because Japan will hold major events, such as the Tokyo Olympics and Paralympic Games in 2021. In addition, it is necessary for us to consider the means of protecting Japan Self Defense Force personnel who have the potential to be exposed to explosions during overseas missions.

Explosion-related injuries are classified into primary blast injury induced by the shock wave, secondary blast injury from penetrating wound caused by flying debris, tertiary blast injury caused by blunt trauma from the blast wind, and quaternary blast injury caused by burns.^5^,^7^,^14^,^21^,^25^,^27^ Among these injuries, fatal damage leading to immediate death is considered to occur due to shock-lung, respiratory arrest, or circulatory failure induced by neurological reflexes such as severe vagal nerve reflex.^11^,^15^

Whether or not a bulletproof vest (body armor) designed to protect the trunk from damage caused by bullets protects against organ damage, such as lung hemorrhage induced by shock waves from explosions, has been the subject of debate.^13^,^18^,^33^ It has not yet been determined whether a bulletproof vest protects against or worsens shock-lung or the neurological reflexes that are considered to be fatal in the super-acute phase after an explosion.

The purpose of this study was to clarify whether or not a bulletproof vest would protect the living body in a pig blast model using a blast tube built at the National Defense Medical College, which is the first such blast tube in Japan.

## Materials and Methods

### Establishment of the Blast Tube

In Japan, we did not have an appropriate model of blast injury because of strict ethical restrictions on animal experiments. Ten years ago, we established a novel small animal model of blast injury using a laser-induced shock wave at the National Defense Medical College (NDMC). Some original articles^10^,^12^,^16^,^22^–^24^ had already been published in international scientific journals; however, these studies only involved small animals (e.g., mice and rats). We needed to establish a medium-sized animal model in order to apply the findings of the studies to human patients.

**Figure 1:**
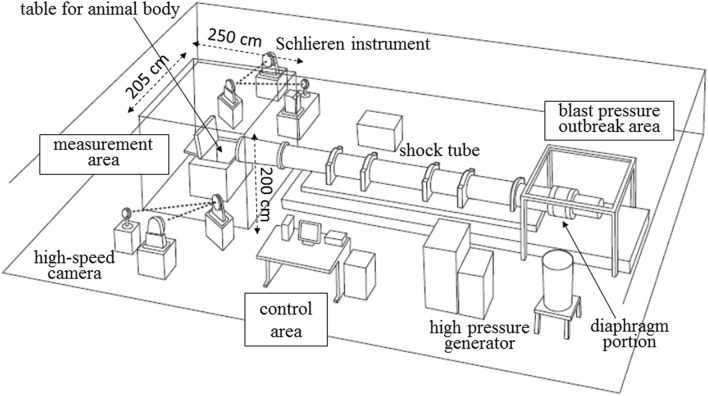
The appearance of the blast tube established at the National Defense Medical College. The blast tube established inside an institute building has a blast pressure-outbreak area, control area, and measurement area. The length of the blast tube is approximately 7.5 m, and the diameter of the outlet window is 40 cm.

**Figure 2:**
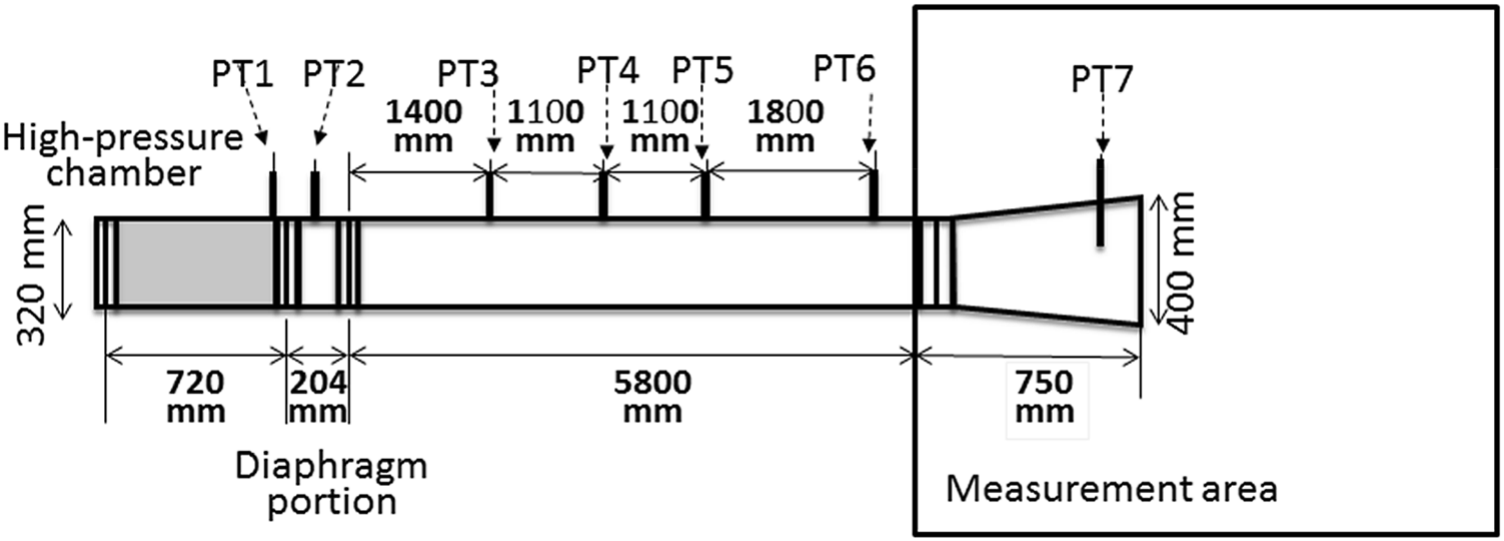
The locations of 7 pressure transducers installed in the shock tube. The static pressure wave is automatically obtained in a computer immediately after metal rupture. PT1, PT2, and PT7 were located in the high-pressure chamber, diaphragm portion, and measurement area, respectively.

**Figure 3:**
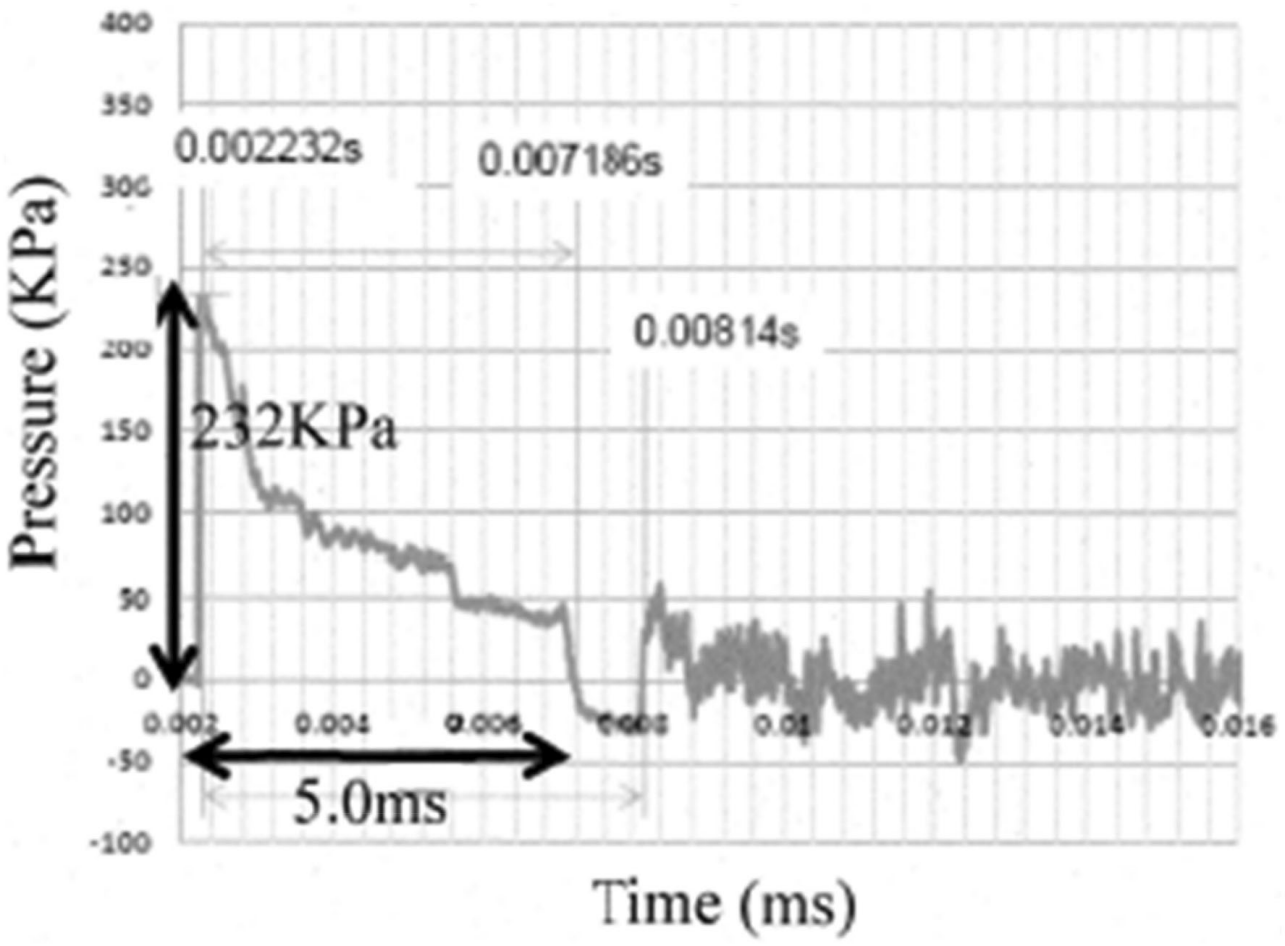
The peak static pressure and duration of action at the outlet window of the shock tube. Preliminary data on the static peak pressure and the duration produced through the outlet window of the shock tube were measured with 3.0 MPa set as the driven pressure.

In 2017, we established a blast tube, which is a device that induces blast injury based on air pressure differences, in conjunction with IHI Corporation (Tokyo, Japan) in the NDMC Research Institute. The development of the blast tube was funded using the budget for Advanced Research on Military Medicine. This blast tube, which was the first of its kind in Japan, enables basic studies on blast injury to be conducted using medium-sized animals. The blast tube established in our institute has a blast pressure-generating area, control area, and measurement area with a Schlieren instrument and a high-speed camera (Fig. 1). The length of the blast tube is about 7.5 m, and the diameter of the outlet window is 40 cm. Data are automatically recorded in a computer system using 7 pressure transducers (PT1–7) inside the blast tube (Fig. 2). The driving pressure of this blast tube can be set to 5.5, 3.0, 1.5, 1.0, or 0.5 MPa. In our experiment, the driving pressure was set to 3.0 MPa based on a previous study by Bass *et al*.^3^ This driving pressure was selected because we predicted that the mortality rate of the animals would be approximately 50% based on the data published by Bass *et al*. and our preliminary data on the static peak pressure and the duration produced through the outlet window of the shock tube (Fig. 3). In addition, we continuously measured the static pressure inside the shock tube using seven pressure transducers (PT1–7) immediately after releasing the driving pressure.

### Animal Experiments

This study was approved by the Animal Ethics Committee of the National Defense Medical College in 2017 and 2018. Seventeen male hybrid pigs (age, 10–12 weeks; mean body weight, 38 kg), were used in these experiments. Before the experiments, each animal was housed in an individual cage in a room with a room 12:12 h light: dark cycle and an ambient temperature of 24 °C. The animals were fed standard laboratory pig chow and had ad libitum access to water. Seventeen pigs were divided into two groups: the body armor group (*n* = 6) and the non-body armor group (*n* = 11).

**Figure 4:**
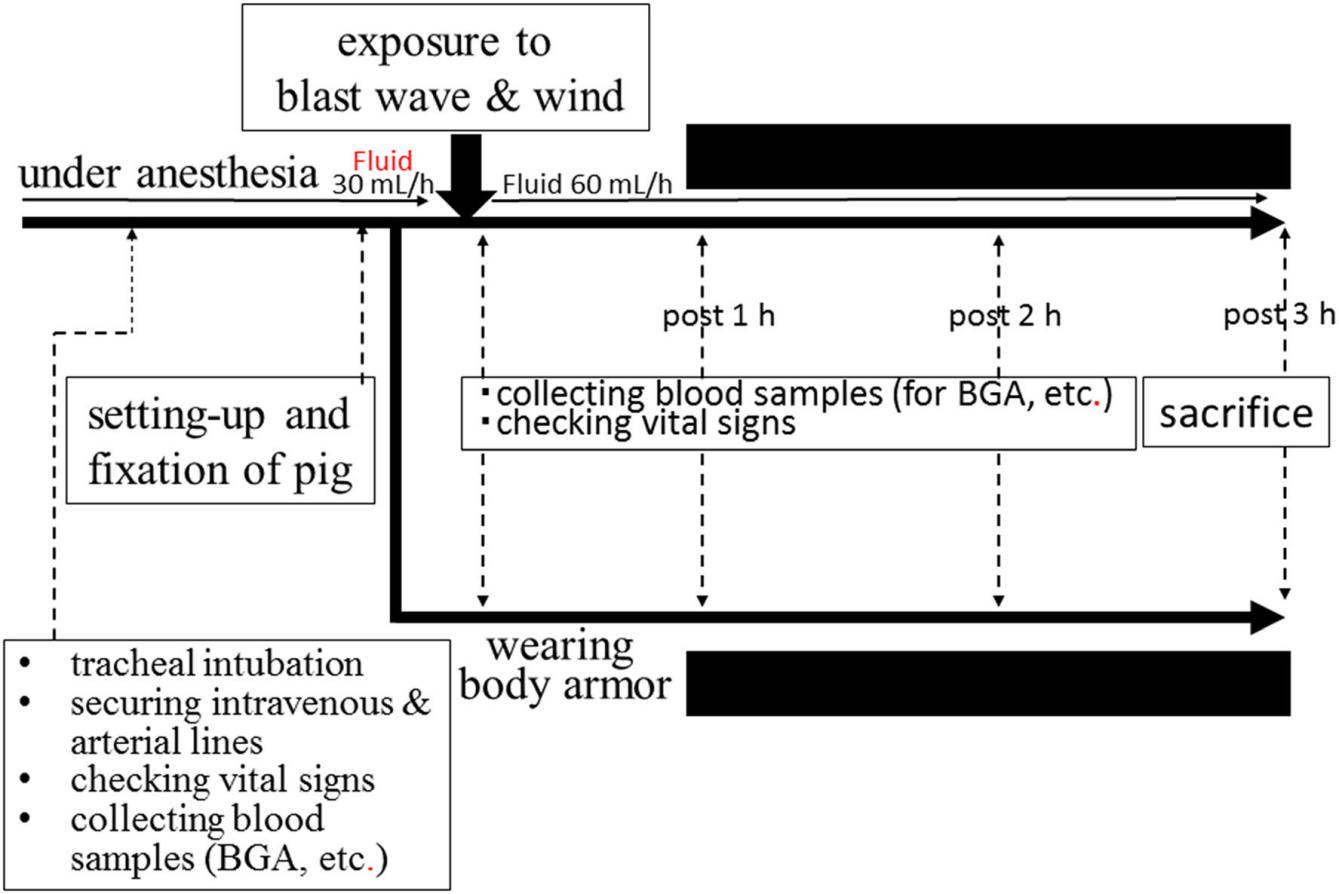
Study design using pigs. We performed tracheal intubation and secured an arterial line via the femoral artery to collect blood samples to measure circulatory blood cell counts and perform blood gas analyses and check the vital signs before the injury. The pig was fixed tightly in the left lateral position on the table in the measurement area. The area from the back of the neck to the upper lumbar back (mainly the upper dorsal back) was then exposed to the blast wave and wind. Immediately after the induction of the blast injury, we observed the respiratory condition and vital signs and then collected heparinized artery blood for a blood gas analysis at 5-minutes post-injury. Each animal had its vital signs checked and blood samples collected every hour, and animals were observed for 3 h after injury. Surviving pigs were sacrificed at 3 h post-injury.

**Figure 5:**
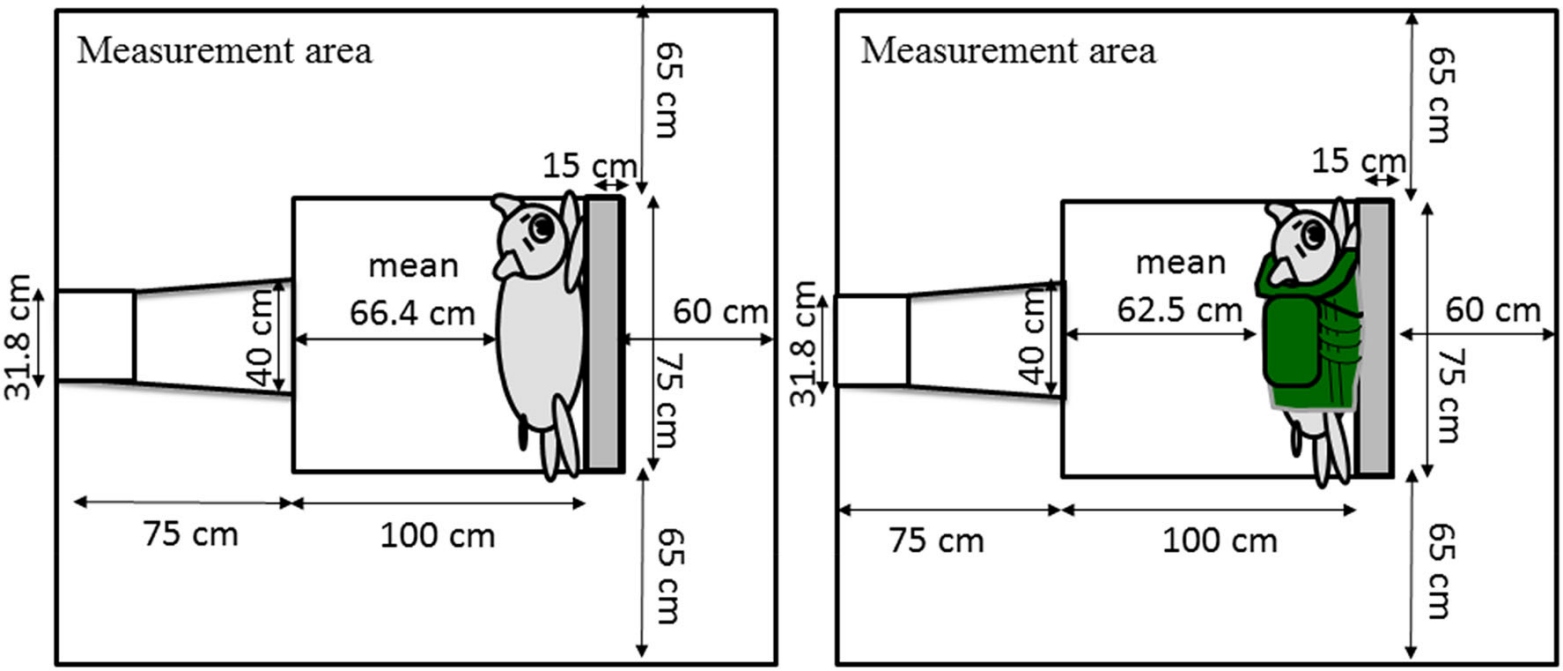
The diagram shows the mean distance from the outlet window of the tube to the dorsal back of the pig in each group, and the position of the pig in the confined space from an upper view.

The design of our animal study is shown in Fig. 4. All animals were anesthetized with ketamine hydrochloride (75 mg/kg sci) and xylazine hydrochloride (45 mg/kg sci). In the animal experiment room, we secured an intravenous line via the subcutaneous auricular vein and administered ketamine hydrochloride (25 mg/kg iv) and xylazine hydrochloride (15 mg/kg iv) every 30 minutes, intravenously. Normal saline was intravenously administered (30 mL/h) to maintain the intravenous line before the injury. In addition, we performed tracheal intubation and secured an arterial line via the femoral artery to collect blood samples (each 5 mL at pre-injury, and 5 min, 1, 2, and 3 h post-injury) to measure circulatory blood cell counts and perform blood gas analyses, and to check the vital signs before the injury. We brought a pig to the room of the blast tube and then fixed it tightly in the left lateral position on the table in the measurement area (i.e., limbs of the pig were tightly fixed by 4 strings and the trunk was fixed by 2 belts to the table). The back of the neck to the upper lumbar back (mainly the upper dorsal back) was then exposed to the blast wave and wind. We adjusted the central axis of the blast tube and the center of the dorsal back in the left lateral position to the same height. The average distance from the outlet window of the blast tube to the dorsal back of the pig was 65 cm (non-body armor group; 65–67 cm, body armor group; 60–65 cm). We showed the mean distance from the outlet window of the tube to the upper dorsal back of a pig in each group, and the position of a pig in the confined space from an upper view (Fig. 5). Immediately after the induction of the blast injury, we observed the respiratory condition and vital signs and then collected heparinized artery blood for a blood gas analysis at 5-min post-injury. The animal was then returned to the animal experiment room and we checked the vital signs and collected blood samples every 1 hour and observed the animals for 3 h after injury. Surviving pigs were sacrificed at 3 h post-injury. Normal saline was intravenously administered (60 mL/h) without fluid resuscitation after injury. At sacrifice, the animals were again anesthetized with ketamine hydrochloride (150 mg/kg iv) and xylazine hydrochloride (90 mg/kg iv), and we macroscopically checked for organ damage in the intrathoracic and intra-abdominal spaces and checked for the existence of apparent fractures of the ribs or chest/lumbar spine.

The body armor worn by the pig was the bulletproof vest 2, which was previously worn by Japan Self-Defense Force (JSDF) personnel. We tried to make the human body armor worn by the pig as tight as possible. The bulletproof vest 2 was designed and deployed for a short period of 3 months in 2003, immediately before Japan Self-Defense Forces were dispatched to Iraq. The material of the bulletproof vest 2 is CORDURA® ballistic nylon, and the vest had ceramic plates (size: 35 × 30 cm) capable of stopping bullets. Body armor with a ceramic plate was used in this study. One ceramic piece was placed in the body armor on the upper dorsal back. The plate covered the heart and partially covered the lungs. The position of the pig fixed in the measurement chamber was almost constant. The anatomical back of the pig was positioned consistently between the conditions; however, there were differences in offset due to the thickness of the body armor. In addition, the body armor type 2 is still being used by some JSDF members.

### Measurements

Blood gas analyses of heparinized arterial blood were conducted using a Vetstat blood gas tester (IDEXX Laboratories, Inc., Japan) to evaluate the respiratory function following blast lung injuries. The circulatory blood count was determined using a multi-item automatic blood cell counter for animals (Sysmex TMC Corp., Tokyo, Japan).

**Figure 6:**
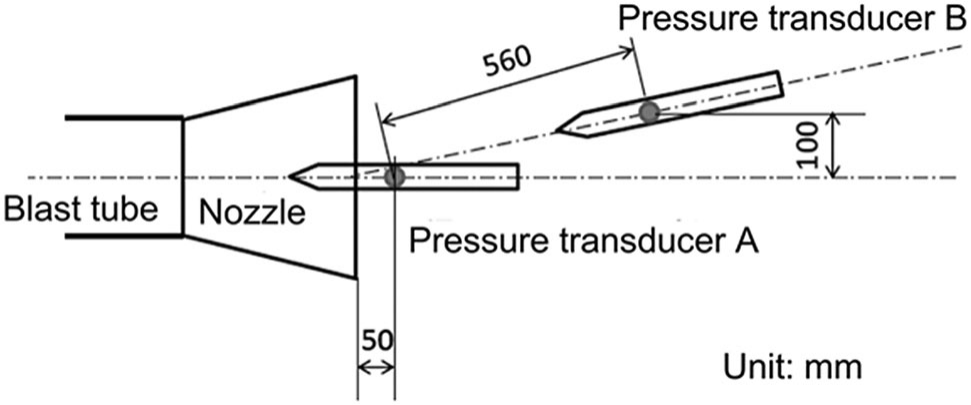
The pressure history caused by the blast tube was confirmed prior to the dose experiment. The locations of pressure transducers in the top view are shown. The location of the dose was approximately 200 mm behind the pressure transducer B. In the dose experiment, the pressure transducers were removed to avoid disturbing the blast wave.

Animals received complex trauma induced by shock and reflected waves. In the test room where an anesthetized pig was fixed in place, we measured and produced calculative pressure images based on the propagation of blast waves. The numerical analysis was performed with a home-made axisymmetric two-dimensional Eulerian code, WAF-2D, in which the weighted average flux (WAF) method was used as the Reimann solver. Shock capturing was implemented by the adaptive mesh method, with an initial mesh size of 5 mm to detect the precise locations of the blast waves. An axisymmetric computational domain comprising a high-pressure chamber of radius 160 mm and length 500 mm, a low-pressure channel of radius 160 mm and length 5800 mm, a nozzle of radius 200 mm (at the exit) and thickness 5 mm, and a test room of radius 1400 mm and length 2780 mm was developed for the analysis. The initial pressure ratio between the high-pressure chamber and the low-pressure channel (P4/P1) was 22.5. The pressure inside the low-pressure channel (P1) was 101.3 kPa, which is the standard atmospheric pressure. The pressure inside the high-pressure chamber (P4) was set to produce shock waves with the same incident shock Mach number obtained from the previous experiment. The pressure history caused by the blast tube was confirmed prior to the dose experiment. Figure 6 shows the locations of the pressure transducers in the top view. The location of the dose was approximately 200 mm behind pressure transducer B in Fig. 6. In the dose experiment, the pressure transducers were removed to avoid disturbing the blast wave.

### Statistical Analyses

All values are expressed as the mean ± standard deviation. The survival rates of the two groups were compared by Fisher’s exact test. The vital signs (systolic blood pressure, pulse, and SpO2), circulatory blood count values (hemoglobin), and blood gas analysis results (PaO2, PaCO2, and pH) were compared between the two groups by a repeated-measures ANOVA. We also examined the association between respiratory arrest and death using Fisher’s exact test. Student’s *t*-test was used for the comparison of continuous variables between two groups. *p* values of < 0.05 were considered to indicate statistical significance.

## Results

The parameters and results of the experiments using 17 pigs (body weight, distance from outlet window to dorsal chest back of each pig, use of body armor, organ damage, and outcome at 3 h post-injury) are summarized in Table 1. There was no significant difference in the body weights of pigs; however, there was a significant difference (*p* < 0.001) in the distance from the outlet window between the body armor and non-body armor groups. It was concluded that the positions of pigs were the same, but that the distances differed due to the thickness of body armor. All pigs injured by the blast wave and wind had lung hemorrhage, and 14 of the 17 pigs had intra-abdominal hemorrhage with splenic injury. All 6 animals in the body armor group and 6 of 11 animals in the control group were alive at 3 h after injury. Although there was not a significant difference in the survival rate between the body armor and non-body armor groups, there was a large difference in the survival rates (body armor vs. non-body armor: 100 vs. 55%). In addition, the organ damage did not appear to differ anatomically between the two groups (Table 1).

**Table 1:** Results of basic research using a swine model.

No.	BW (kg)	Wearing of body armor	Remarks on organ damage (by sacrifice, etc.)	Respiratory arrest	Outcome
①	36.5	No	LH, Hemothorax, Splenic injury, IAH	Yes	Death
②	34.5	No	LH, Splenic injury, IAH	Yes	Death
③	39	No	LH, Splenic injury, IAH	Yes	Death
④	39	No	LH, Hemothorax, Splenic injury, IAH	Yes	Death
⑤	40	No	LH, Hemothorax, Splenic injury, BR, IAH	Yes	Death
⑥	40	No	LH, Splenic injury, IAH	No	Survival
⑦	39	No	LH, Hemothorax	No	Survival
⑧	37.5	No	LH, Hemothorax, Splenic injury, IAH	No	Survival
⑨	37	No	LH, Splenic injury, IAH	Yes	Survival
⑩	48	No	LH, Splenic injury, IAH	No	Survival
⑪	36	No	LH, Splenic injury, IAH	No	Survival
⑫	36	Yes	LH	No	Survival
⑬	38	Yes	LH, Splenic injury, IAH	No	Survival
⑭	36	Yes	LH, Splenic injury, IAH	No	Survival
⑮	39	Yes	LH, Splenic injury, IAH	Yes	Survival
⑯	35.5	Yes	LH	No	Survival
⑰	39.5	Yes	LH, Splenic & Liver injury, BR, IAH	Yes	Survival

*LH* lung hemorrhage, *IAH* intra-abdominal hemorrhage, *BR* bladder rupture

**Figure 7:**
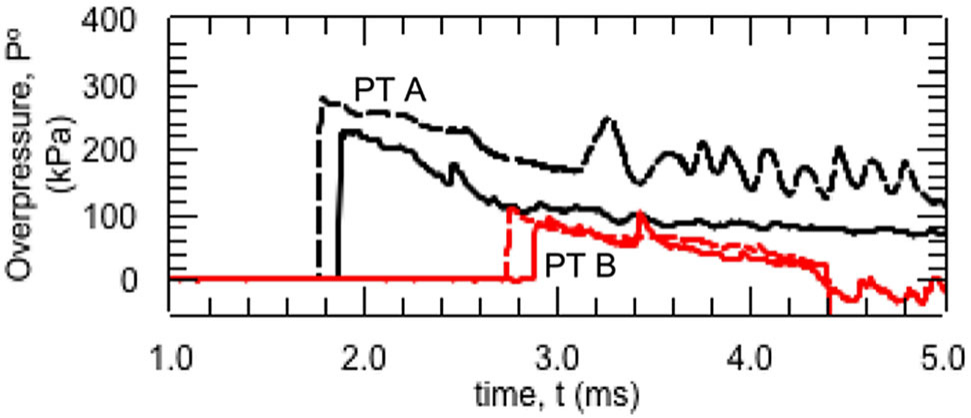
The comparison between the pressure history measured with pressure transducers (PT A and PT B), and one obtained by numerical analyses are shown. The black solid and broken lines indicate the pressure history at PT A, the red solid and broken lines indicate the pressure history at PT B. The solid line indicates the results of the experiment, the broken line indicates the results of the numerical analysis. The values for the arrival time, peak overpressure, and duration of the blast wave were similar.

The comparison between the pressure history measured with pressure transducers (PT A and PT B), and a comparison by numerical analyses are shown in Fig. 7. The black solid and broken lines indicate the pressure history at PT A, while the red solid and broken lines indicate the pressure history at PT B. The solid line indicates the results of the experiment; the broken line indicates the results of the numerical analysis. The values for arrival time, peak overpressure, and duration of the blast wave were similar. On the other hand, the detailed shape of the numerical results was slightly different from that of the experimental results because the fixtures of the pigs were not simulated in this numerical simulation. However, even though slight differences were found in the pressure histories, the numerical results showed good agreement with the experiments.

**Figure 8:**
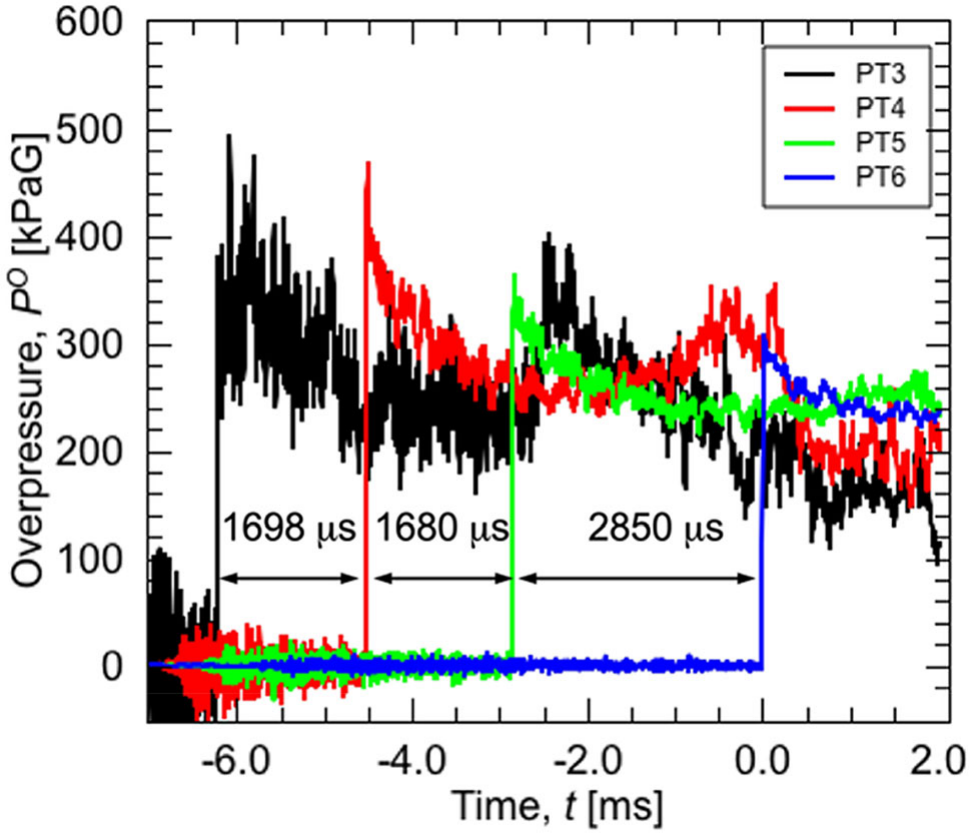
Pressure transducers (PT3, 4, 5, and 6) were installed to detect the passage of the shock waves and overpressure at the distances shown in the figure. The typical arrival time and overpressure provided by shock waves in the case of operation at 3.0 MPa in pig No. 10 is shown.

In the numerical analysis, the initial pressure of the high-pressure chamber was set to 2.3 MPa, based on the Mach number obtained from the experiment. Figure 2 shows the location of the pressure transducers installed in the shock tube. Pressure transducers (PT3, 4, 5, and 6) were installed to detect the passage of shock waves and overpressure at the distances shown in the figure. Figure 8 shows the typical arrival time and overpressure provided by shock waves in the case of operation at 3.0 MPa in pig No. 10. One can calculate the shock wave velocity and shock Mach number (Ms) by dividing the travel distance by the travel duration and speed of sound. In the case of the 3.0 MPa condition, the Ms was 1.859, which is equivalent to 2.3 MPa of initial pressure in a high-pressure chamber, according to the simple shock tube theory, which is used for the analysis of fluid dynamics in shock tube operation. The difference between the initial pressure given by the simple shock tube theory and the present experiment is explained as follows. The simple shock tube theory assumes that the diaphragm is single and will disappear instantaneously and completely. On the other hand, the diaphragm for the experiment was a double diaphragm, required a finite duration for rupture, and ruptured incompletely. (b) The simple shock tube theory does not assume friction and heat exchange at the wall of the shock tube; however, the experiment causes these phenomena. Therefore, for the numerical calculation based on the simple shock theory, it is necessary to set an initial pressure that will provide the same Ms value as the real experiments provide.

**Figure 9:**
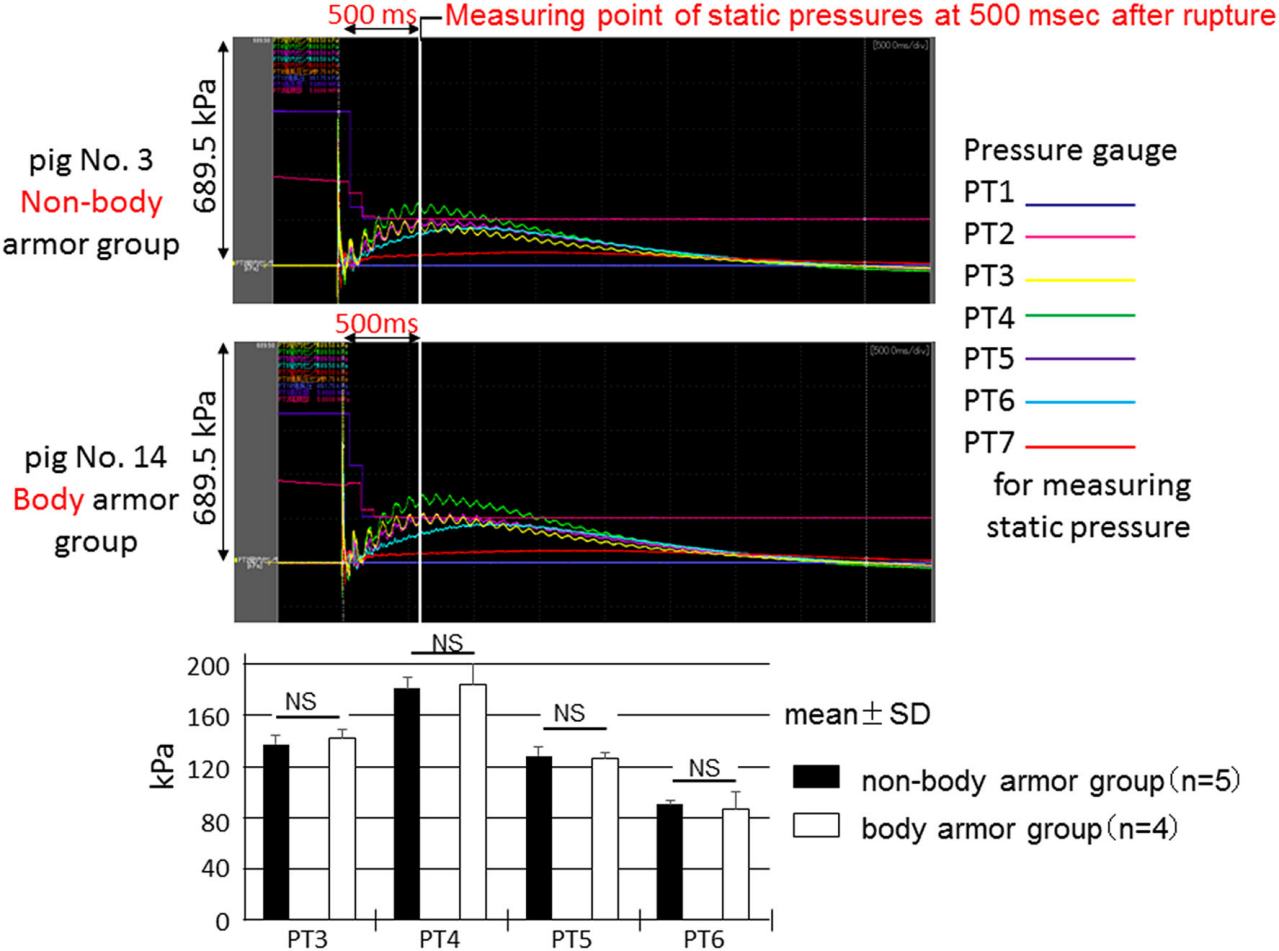
Static pressure waves measured using 7 pressure transducers that were located in the shock tube for pigs No. 3 and No. 14 are shown. Pig No. 3 was in the non-body armor group, while pig No. 14 was in the body armor group. The static pressure waves that were induced by the blast waves or winds did not seem to differ between the two pigs. In addition, we compared static pressures, which were measured by PT3, 4, 5, and 6 inside the shock tube using the pressure at 500 msec after the metal membrane rupture, between the two groups. The data obtained in animals No. 2, 3, 6, 10, and 11 in the non-body armor group and No. 12, 14, 15, and 17 in the body armor group were used in these statistical analyses. The other animals’ data were not measured. Although there were missing data in the analyses, the static pressure inside the tube did not significantly differ between the two groups.

We showed static pressure waves measured using 7 pressure transducers, that were located in the blast tube as shown in Fig. 2, for pigs No. 3 and 14. Pig No.3 was in the non-body armor group, while pig No. 14 was in the body armor group. The static pressure waves that were induced by the blast waves and winds did not seem to differ between the two pigs (Fig. 9). In addition, we compared the static pressures, which were measured by PT3, 4, 5, and 6 inside the shock tube using the pressure at 500 msec immediately after releasing the driving pressure, between the groups. The data obtained in animals No. 2, 3, 6, 10, and 11 in the non-body armor group and No. 12, 14, 15, and 17 in the body armor group were used in these statistical analyses. The static pressures inside the shock tube in the other animals were not measured. Although there were missing data in the analyses, the static pressure inside the tube did not significantly differ between the two groups.

Eight pigs in the two groups suffered respiratory arrest immediately after exposure to the blast wave and wind; 3 pigs spontaneously recovered from respiratory arrest and survived for 3 h after injury. As a result, all pigs in the body armor group survived for 3 h. The association between respiratory arrest and death was statistically significant (Fisher’s exact test; *p* = 0.009). Respiratory arrest immediately after exposure to the blast wave and the wind was considered to influence the outcomes in our pig model (Table 2).

**Table 2:** Relationship between respiratory arrest and outcome

	Outcome		
	Survived	Dead	Total
Respiratory arrest			
No	9	0	9
Yes	3	5	8
Total	12	5	17

The association between respiratory arrest and death was significant (Fisher’s exact test; *p* = 0.009). Eight pigs had respiratory arrest immediately after exposure to the blast wave and wind in two groups, and three of the animals spontaneously recovered from respiratory arrest and survived for three hours after injury.

**Figure 10:**
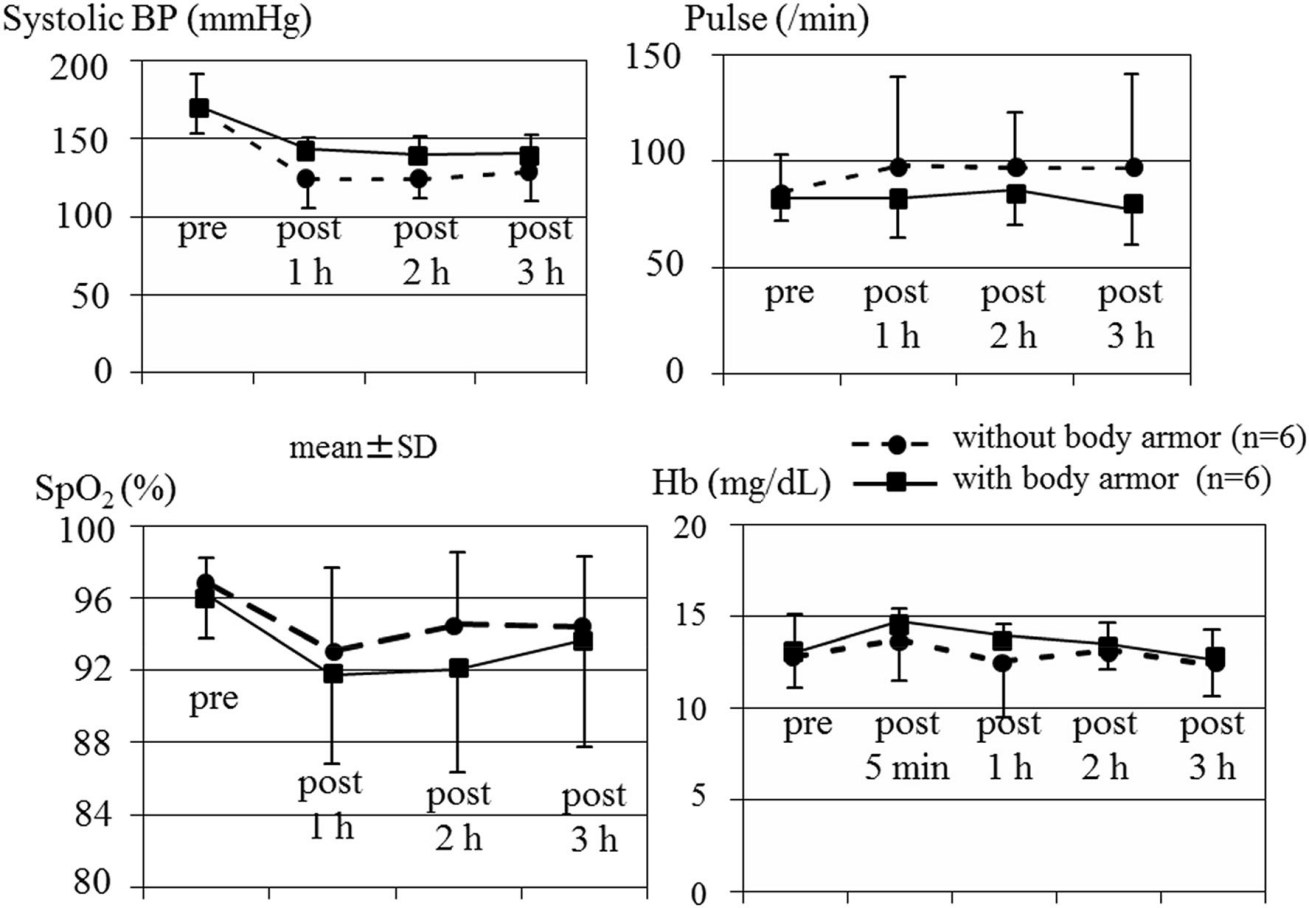
Changes in the vital signs in two groups with and without body armor among surviving pigs. The changes in the systolic blood pressure, pulse rate, and SpO_2_ did not differ markedly between the two groups to a statistically significant extent. Hemoglobin values in the two groups with and without body armor among surviving pigs. The changes in these values did not differ significantly between the two groups.

**Figure 11:**
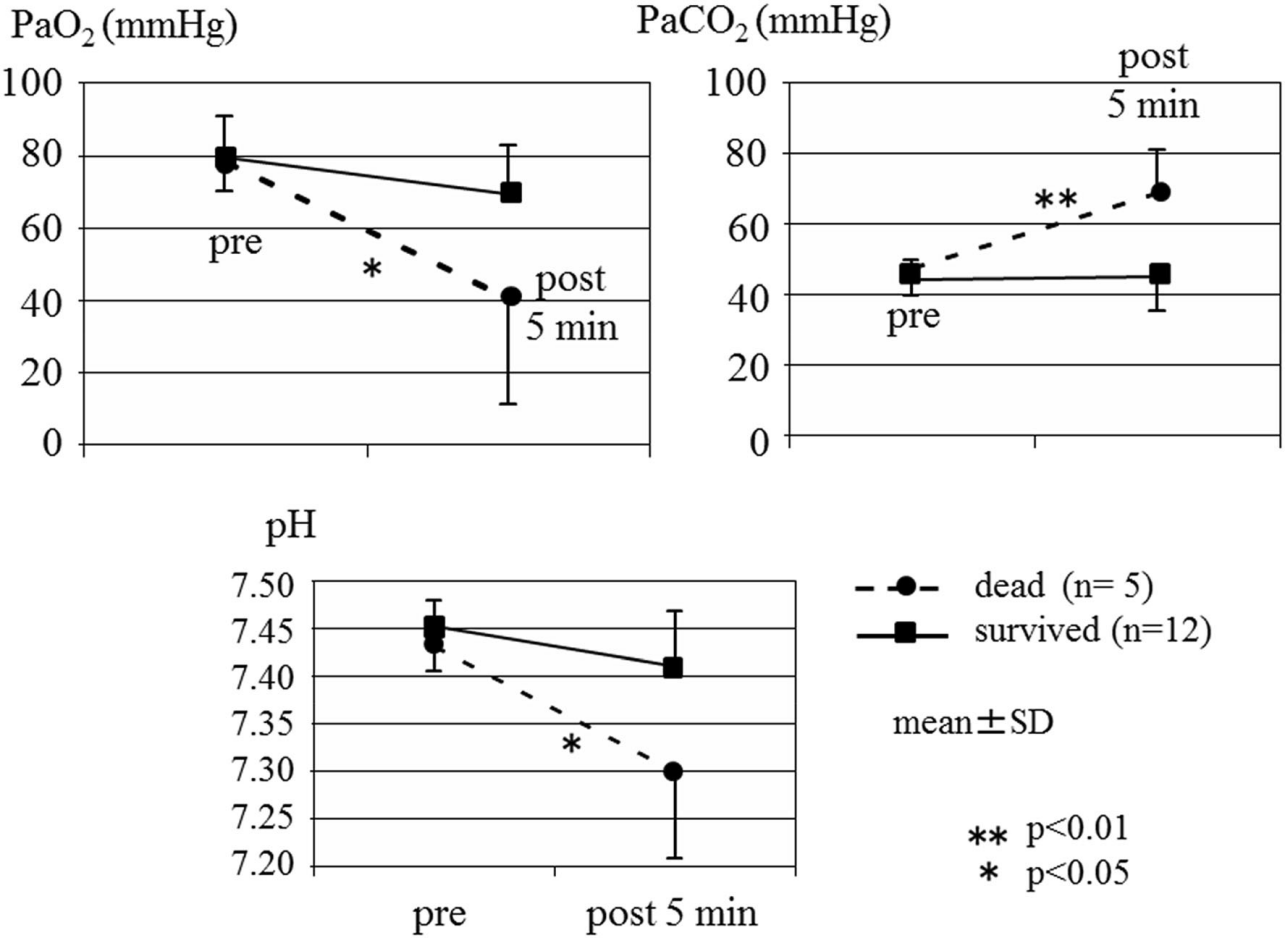
Results of blood gas analyses in surviving and dead pigs. Significant differences in the blood gas analysis results (PaO2, PaCO2, and pH) were noted between the surviving and dead pigs.

The changes in the vital signs, hemoglobin values, and blood gas analyses results of the surviving pigs with and without body armor are shown in Fig. 10, respectively. These parameters did not differ between the two groups to a statistically significant extent. In contrast, Fig. 11 demonstrates significant differences in the blood gas analysis results (PaO2, PaCO2, and pH) of the surviving and dead pigs.

**Figure 12:**
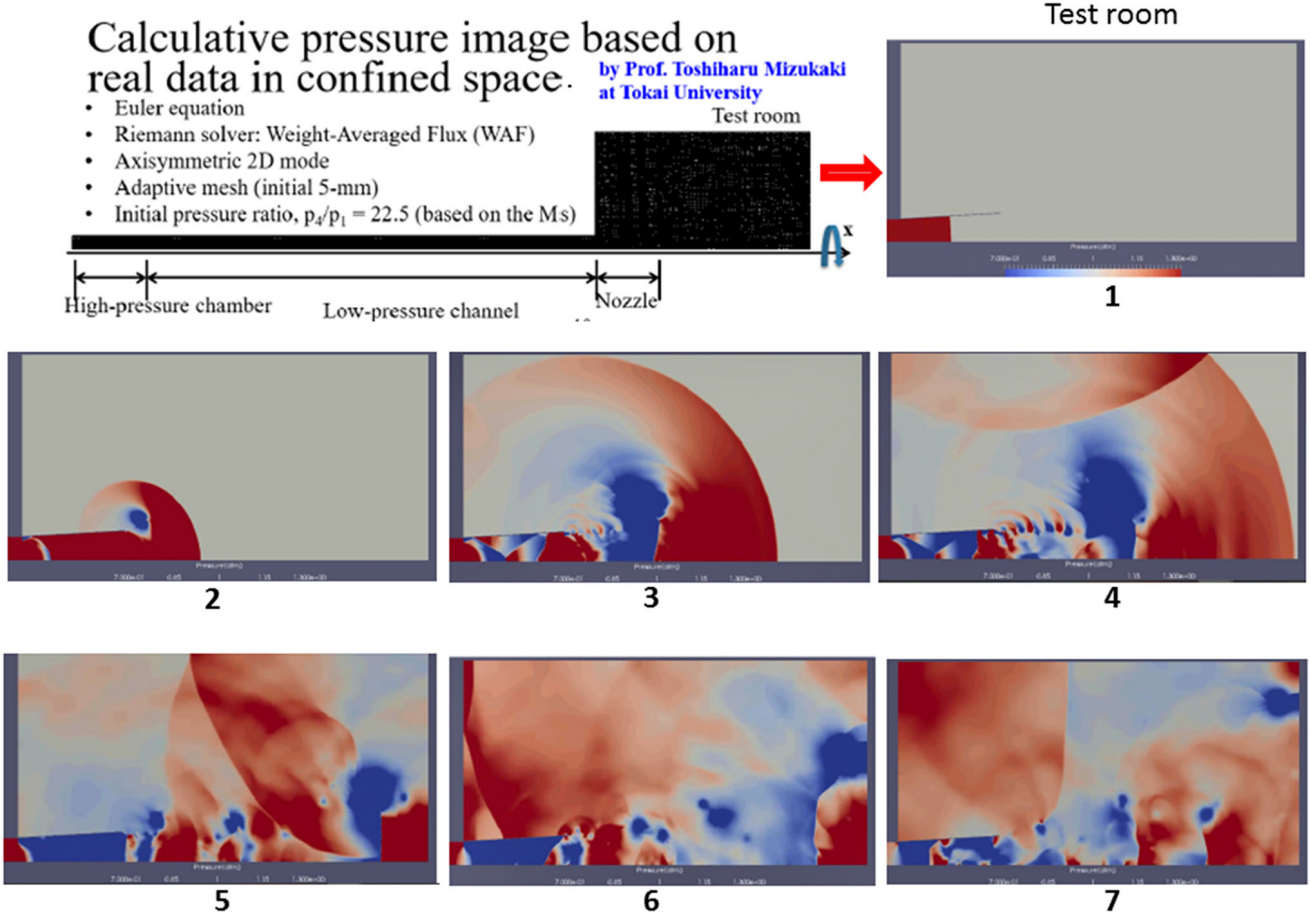
Numerical results concerning the propagation of blast waves inside the test room. This figure shows a computer-generated pressure image created based on the real sizes of the shock tube and test room and the measured pressures in a confined space. (1)–(7) show the time-resolved development of the blast waves discharged from the nozzle into the test room. The color indicates the overpressure: red indicates ≥ 30.4 kPa, and blue indicates ≤ − 30.4 kPa, generated by the blast waves. Finally, the overpressure inside the test room increased (in the entire region) due to the multiple reflected blast waves.

Figure 12 shows a computer-generated pressure image created based on the real sizes of the shock tube and test room and the measured pressures in a confined space. Figure 12(1)–(7) shows the time-resolved development of the blast waves discharged from the nozzle into the test room. The color indicates the overpressure: red indicates ≥ 30.4 kPa (130% of the initial pressure, 101.3 kPa), and blue indicates ≤ − 30.4 kPa (70% of the initial pressure), generated by the blast waves. As shown in Fig. 8(1), at *t* = *t*0, the incident shock wave propagated inside the low-pressure channel arrived at the middle section of the nozzle. As shown in Fig. 8(2), at *t* = *t*0 + 1.00 ms, the incident shock wave was discharged from the nozzle, and the strong shock wave along the center axis propagated into the test room, generating vortexes beside the nozzle (indicated by blue). At *t* = *t*0 + 3.35 ms, the diffracted shock waves reached the wall of the test room (Fig. 8(3)). At *t* = *t*0 + 4.75 ms, the shock waves reflected from the wall of the test room appeared, and the incident shock waves reached the right side (of the wall) of the test room (Fig. 8(4)). At *t* = *t*0 + 10.00 ms, the shock waves reflected from the right side (of the wall) interfered with the shock waves reflected from the upper side of the test room and the jet of the nozzle (Fig. 8(5)). As shown in Fig. 8(6), at *t* = *t*0 + 15.00 ms, the shock waves were reflected from the right side of the wall toward the opposite wall of the test room, experiencing interference with the reflected waves from the upper wall and jet. Finally, the overpressure inside the test room increased (in the entire region) due to the multiple reflected blast waves. According to the numerical results, in the test room, the incident shock waves directly produced a strong impulse on the test object followed by multiple impacts caused by a large number of reflected shock waves.

## Discussion

There are have been many recent studies in the blast injury field. In particular, a large number of articles have investigated mild blast traumatic brain injury induced by blast shock waves.^28^,^30^ In contrast, the present study focuses on acute physiological changes, including respiratory arrest and blast lung. We chose the medium-sized pig as an experimental animal for investigating human blast injury. Pigs have short limbs and a relatively large trunk; thus, pigs of approximately 40 kg in body weight are suitable for simulating trunk damage in human adults. Furthermore, humans and pigs are anatomically similar, and we studied blast injuries induced by a blast tube, which was the first of its kind in Japan. This enables basic studies on blast injury to be conducted using medium-sized animals. The test configuration of a large specimen positioned outside the shock-tube exit likely created localized loading on the pig, which was centered on the protective armor. This would not be expected in the real world, and the animal anatomy and the position and restraint of the pig in a confined space—with respect to the applied loading—became particularly important (Figs. 1, 5, and 12).

In 1957, the importance of the vagal reflex and autonomous nervous system response to blast wind and shock waves was demonstrated by Clemedson.^8^ They showed that the neurological reflex immediately after blast exposure induced respiratory arrest, slow breathing, hypotension, and bradycardia. Apnea was rare in rabbits with vagotomy after exposure to high shock waves using a blast tube.^8^ When the pressure wave propagates in the body, the pressure inside the organs increases,^9^ and increased pressure in the lung causes instantaneous lung hyperinflation. Then, the juxtacapillary J-receptors located in the alveolar interstitium are stimulated,^17^ inducing the vagal reflex. Pressure receptors on the walls and trabeculae of the left ventricle can activate C-fiber afferents to cause bradycardia, which in turn reduces myocardial contractility and deepens arterial hypotension.^6^ However, some mechanisms in the neurological reflexes in settings such as injuries to the brain stem and the medulla oblongata located in the posterior high neck remain unclear, as does association with blast-induced neurotrauma (BINT). At the very least, our study revealed that in pigs with respiratory arrest, the only factor that differed between survivors and non-survivors was the spontaneous recovery of breathing. Although we did not evaluate it in this study, vital signs (blood pressure, pulse rate, arterial oxygen saturation, and respiratory rate) can be demonstrated using video recordings or similar modalities immediately after exposure of an animal to the shock wave and blast wind. In future studies, CT will be used to evaluate the extent of blast lung damage using special software programs to prove that the severity of lung damage does not match the immediate physiological changes. I intend to conduct more studies to obtain data proving the neurological mechanism that induces respiratory arrest and similar related events.

In this study, pigs with bulletproof vests (body armor) had higher survival rates than pigs without bulletproof vests. Moreover, all of the dead pigs in this study had respiratory arrest immediately after exposure to the blast wave and wind. Only pigs with spontaneously recovered breathing survived for 3 h. All of the pigs that did not recover from respiratory arrest died within one hour of injury. Thus, it was clear that the occurrence of respiratory arrest, which appears to be a neurological reflex that occurs immediately after injury, is significantly involved in the 3-h outcome. We hypothesized that the body armor reduced the incidence of neurological respiratory arrest and protected the pig’s life by lessening the insult induced by the blast wave and wind. At any rate, the body armor significantly improved the survival rate, and it was considered to be beneficial to the living body in terms of life support in the super-acute phase after an explosion.

Phillips *et al*. reported that wearing an army ballistic jacket was not beneficial for protecting against shock wave damage to the chest.^18^ Their study involved basic blast injury experiments using sheep. Although there were numerous differences in the conditions of their study and our own, they reached the opposite conclusions with regard to the lifesaving effect of wearing a bulletproof vest on blast injury. The length of the blast tube used by Phillips et al. was 36.6 m, and they applied shock waves and blasts to the right side of sheep placed in an open field. When the peak pressure was 420 kPa, which was the highest, 5 out of 6 sheep with bulletproof vests died, while only 2 out of 6 sheep in the non-body armor group died. According to Phillips et al., it was considered that the pressure in the vest increased and that blast-lung was exacerbated. However, the results of the present study contradict the suggestion that wearing a bulletproof vest exacerbates blast-lung. The organ damage induced by a blast wave in a confined space may be worse than that induced in an open space due to wave reflection. The ceramic back plate in our body armor may only protect the pig’s lungs and heart from the initial blast load. The reflected waves shown in Fig. 12 may have also severely damaged the lungs and heart. We believe that wearing body armor had beneficial effects in the super-acute phase because it reduced the incidence of respiratory arrest and increased the survival rate.

In this study, we investigated mortality and organ injury when blast waves and wind were applied from the back of the neck to the upper lumbar back centering on the upper dorsal back during an explosion in a confined space. In the absence of a respiratory arrest, all pigs survived for 3 h after injury without resuscitation. When we raised the driving pressure of the blast tube to 5.5 MPa, the pigs exhibited not only respiratory arrest but also reduced blood pressure and cardiac arrest due to ventricular fibrillation immediately after injury (data not shown). Thus, we suggest that surviving is possible if the respiratory and cardiac arrest does not occur immediately after an explosion and lifesaving care and damage control surgery are received as soon as possible in instances of exposure to driving pressure from a blast tube at 3.0 MPa. Wearing body armor may be beneficial to saving lives if an explosion occurs.

### Limitations

The present study was associated with some limitations. First, this study simulated an explosion in a confined space, organ injury is likely to be more severe than that in an open space. Second, the organ damage and mortality may differ depending on the animal posture, body parts, and angle of exposure to the blast wave and wind. Furthermore, positions and materials such as animal tables and rear fixtures may have had protective effects. Third, animal species differed from previous studies. Moreover, there are anatomical and physiological differences between humans and animals. There is also a study using human corpses^4^; however, in the present study, we conducted blast injury experiments using medium-size animals. Finally, the fit of the body armor to the trunk was not ideal because it was designed for humans. Even so, the body armor had a protective effect against shock waves.
